# Evaluation of an in-house indirect enzyme-linked immunosorbent assay of feline panleukopenia VP2 subunit antigen in comparison to hemagglutination inhibition assay to monitor tiger antibody levels by Bayesian approach

**DOI:** 10.1186/s12917-020-02496-z

**Published:** 2020-08-06

**Authors:** Chanakan Areewong, Amarin Rittipornlertrak, Boondarika Nambooppha, Itsarapan Fhaikrue, Tawatchai Singhla, Chollada Sodarat, Worapat Prachasilchai, Preeyanat Vongchan, Nattawooti Sthitmatee

**Affiliations:** 1 Graduate School of Veterinary Science, Faculty of Veterinary Medicine, Chiang Mai University, Chiang Mai, 50100 Thailand; 2 Tiger Kingdom, Mae Rim, Chiang Mai, 50180 Thailand; 3 Department of Medical Technology, Faculty of Associated Medical Sciences, Chiang Mai University, Chiang Mai, 50200 Thailand; 4 Excellence Center in Veterinary Bioscience, Chiang Mai University, Chiang Mai, 50100 Thailand; 5 Department of Veterinary Bioscience and Veterinary Public Health, Faculty of Veterinary Medicine, Chiang Mai University, Chiang Mai, 50100 Thailand

**Keywords:** Bayesian model, Capsid protein VP2, Feline panleukopenia virus, Hemagglutination inhibition assay, Indirect ELISA, Tiger (*Panthera tigris*)

## Abstract

**Background:**

Feline panleukopenia virus (FPV) is an etiologic pathogen of feline panleukopenia that infects all members of Felidae including tigers (*Panthera tigris*). Vaccinations against FPV among wild felid species have long been practiced in zoos worldwide. However, few studies have assessed the tiger immune response post-vaccination due to the absence of a serological diagnostic tool. To address these limitations, this study aimed to develop an in-house indirect enzyme-linked immunosorbent assay (ELISA) for the monitoring of tiger antibody levels against the feline panleukopenia vaccine by employing the synthesized subunit capsid protein VP2. An in-house horseradish peroxidase (HRP) conjugated rabbit anti-tiger immunoglobulin G (IgG) polyclonal antibody (HRP-anti-tiger IgG) was produced in this study and employed in the assay. It was then compared to a commercial HRP-conjugated goat anti-cat IgG (HRP-anti-cat IgG). Sensitivity and specificity were evaluated using the Bayesian model with preferential conditional dependence between HRP-conjugated antibody-based ELISAs and hemagglutination-inhibition (HI) tests.

**Results:**

The posterior estimates for sensitivity and specificity of two indirect ELISA HRP-conjugated antibodies were higher than those of the HI test. The sensitivity and specificity of the indirect ELISA for HRP-anti-tiger IgG and HRP-anti-cat IgG were 86.5, 57.2 and 86.7%, 64.6%, respectively, while the results of the HI test were 79.1 and 54.1%. In applications, 89.6% (198/221) and 89.1% (197/221) of the tiger serum samples were determined to be seropositive by indirect ELISA testing against HRP-anti-tiger and HRP-anti-cat, respectively.

**Conclusion:**

To the best of our knowledge, the specific serology assays for the detection of the tiger IgG antibody have not yet been established. The HRP-anti-tiger IgG has been produced for the purpose of developing the specific immunoassays for tigers. Remarkably, an in-house indirect ELISA based on VP2 subunit antigen has been successfully developed in this study, providing a potentially valuable serological tool for the effective detection of tiger antibodies.

## Background

Feline panleukopenia, also referred to as feline distemper or feline infectious enteritis, is a contagious disease that is common among domestic cats (*Felis catus*) and other Felidae with high rates of morbidity and mortality [1–4]. Feline panleukopenia virus (FPV) is an etiologic agent of the disease. The mortality rate can range from 25 to 100% depending on the severity of the clinical signs. However, the current prevalence of subclinical infections is unknown [5]. Cats of all ages can be infected with FPV, but young animals are the most susceptible. Previous investigations have reported incidences of fatal FPV infections among captive felids. Duarte et al. (2009) [6] reported on the death of a white tiger (*Panthera tigris*) and an African lion (*Panthera leo*) from FPV infection at the Lisbon zoo in Portugal. Dissanayake et al. (2017) [7] reported on the death of an unvaccinated Bengal tiger cub (*Panthera tigris tigris*) and severe illness in an unvaccinated leopard cub (*Panthera pardus*) at a zoological garden in Sri Lanka, both of which were caused by FPV. These incidences indicated that tigers, which are the largest known cat species, appear to be particularly susceptible to FPV infection. Moreover, feline panleukopenia in tigers should be of significant concern because tigers are an endangered species according to the International Union for Conservation of Nature’s (IUCN) Red List of Threatened Species [8].

An effective vaccination policy is the most important strategy for the prevention of FPV infection [5]. Vaccinations against FPV have been a routine element of feline preventative medicine for the past 40 or more years [9]. Despite the frequent use of effective vaccines in domestic cats, there are no vaccines and vaccination programs that have been approved for use in non-domestic felids. Importantly, vaccination policies have long been practiced in zoos worldwide. Therefore, the application of vaccines and vaccination programs for cats are now being suggested for use among wild felids. In practice, modified live vaccines (MLV) are commonly used on domestic cats. However, inactivated vaccines are recommended for use among tigers and other wild felids that are held in captivity [10, 11]. This is due to the potential risk of inducing a range of diseases, or the fact that they may lead to mutations when MLVs are used among species upon which the vaccines have never been tested. Many zoological parks, including some in Thailand, have also used inactivated vaccines to prevent FPV infection in tigers. However, the effectiveness of using a cat’s inactivated FPV vaccine on captive tigers is unknown. Additionally, suitable vaccine protocols have not yet been clarified due to the lack of an established specific serological method for the evaluation of tiger antibodies post vaccination.

Generally, the detection of antibodies against FPV is commonly achieved by the hemagglutination-inhibition (HI) test or enzyme-linked immunosorbent assay (ELISA). The recombinant capsid protein or viral protein 2 (VP2) protein of FPV has been used as a coating antigen for ELISA in order to detect the level of the FPV-specific immunoglobulin G (IgG) in cats [12]. The capsid protein VP2, a major structural protein of FPV, is a well characterized gene that has been widely used for phylogenetic analysis because it encodes the major protein that determines the host’s range, along with the relevant viral pathogenicity and immune response [4, 6]. Therefore, to address the limitations of the present serological diagnostic tools for tigers, this study aimed to develop an in-house indirect ELISA test kit for the monitoring of tiger antibody levels against the feline panleukopenia vaccine by employing the VP2 protein as a coating antigen. An in-house horseradish peroxidase (HRP) conjugated rabbit anti-tiger IgG polyclonal antibody was also employed in this study as a secondary antibody. Notably, this in-house indirect ELISA test kit will be particularly helpful in laboratory investigations involving disease surveillance of FPV among tigers.

## Results

### Generation and binding capability of HRP-conjugated rabbit anti-tiger IgG antibody

**Fig. 1:**
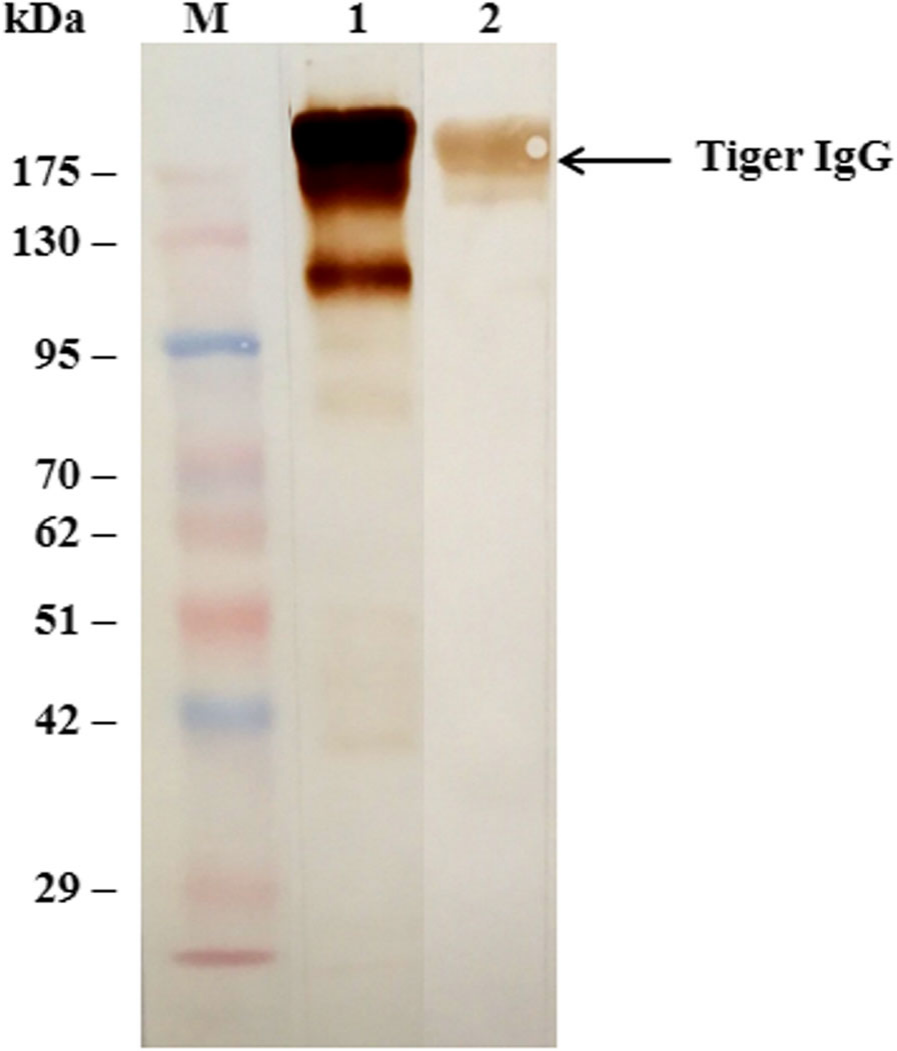
Western blot analysis of binding capability of HRP-conjugated IgG antibody against Bengal tiger IgG. Lane M: Protein marker; Lane 1: HRP-conjugated goat anti-cat antibody, Lane 2: HRP-conjugated rabbit anti-tiger IgG antibody

The purified rabbit anti-tiger IgG was conjugated with HRP. The binding capability of HRP-conjugated rabbit anti-tiger IgG antibody was evaluated by western blotting analysis. The results showed the reactive protein bands at approximately 170 kDa under non-reducing conditions as the expected size of the tiger IgG antibody, as well as of the HRP-conjugated goat anti-cat IgG antibody (Fig. 1). The results demonstrated the capturing ability and specificity of the HRP-conjugated antibody to purified tiger IgG antibody.

### Optimal concentration of in-house indirect ELISA reagents and cut-off value

The signal to noise (S/N) ratio between the optimal density (OD) value of the vaccinated and non-vaccinated sera at 450 nm is shown in Table 1. The optimal conditions for the indirect ELISA were 1 ng of capsid protein VP2 in 100 μl of the coating buffer as a coating antigen. A dilution of 1:200 for the tiger serum with 1:1000 HRP-conjugated goat anti-cat IgG antibody, and a dilution of 1:100 for the tiger serum with 1:2000 HRP-conjugated rabbit anti-tiger IgG antibody, were selected for use as the primary and secondary antibodies in this study.

**Table 1:** Optimal serum dilution and HRP-conjugated antibody dilution reacted with 1 ng/100 μl of coating buffer

HRP-Conjugated antibody	Serum dilution / HRP dilution	S/N ratio				
		1:100	1:200	1:300	1:400	1:500
Anti-cat	1:1000	2.460	**3.095**^**a**^	3.508	3.679	3.951
	1:2000	2.310	2.724	2.921	3.085	3.122
	1:3000	2.350	2.641	2.959	2.949	2.983
	1:4000	2.300	2.730	2.749	2.733	2.826
Anti-tiger	1:1000	3.812	2.736	2.651	2.235	1.949
	1:2000	**3.160**^**a**^	2.600	2.134	1.825	1.732
	1:3000	2.577	2.132	1.968	1.649	1.603
	1:4000	2.651	2.283	2.000	1.582	1.637

^a^ Represents suitable S/N ratio obtained from this study

Average OD values at 450 nm and standard deviations of HRP-conjugated goat anti-cat antibody were 0.265 and 0.043, respectively. Thus, the cut-off point of the indirect ELISA using HRP-conjugated goat anti-cat antibody was 0.40. Additionally, the average OD value at 450 nm of the HRP-conjugated rabbit anti-tiger antibody was 0.069, and the standard deviation value was 0.006. Therefore, the cut-off point of the indirect ELISA using HRP-conjugated rabbit anti-tiger antibody was 0.09.

### Seroprevalence of antibody titers against feline panleukopenia vaccine in tigers

The results of an indirect ELISA using two different HRP-conjugated antibodies where compared to the HI test and are shown in Table 2. Considering the HI titer at 1:20, the seropositive value of tiger antibodies against FPV vaccine was 80% (178/221). In contrast, the seropositive value measured by in-house indirect ELISA tests with neither anti-tiger nor anti-cat antibody were 89.6% (198/221) and 89.1% (197/221), respectively.

**Table 2:** Comparison of indirect ELISA test and HI test to evaluate the seroprevalence against FPV vaccine

Diagnostic tests	HI positive		HI negative		Total
	ELISA anti-cat positive	ELISA anti-cat negative	ELISA anti-cat positive	ELISA anti-cat negative	
ELISA anti-tiger positive	164	1	32	1	198
ELISA anti-tiger negative	1	12	0	10	23
Total	165	13	32	11	**221**

### Sensitivity and specificity of an in-house indirect ELISA

The posterior estimates for sensitivity (Se) and specificity (Sp) of each test and the prevalence of tiger antibodies against the FPV vaccine are shown in Table 3. The Se values for anti-cat ELISA, anti-tiger ELISA and the HI test were found to be higher than the prior median values of 86.7% [95% posterior probability interval (PPI) = 82.0–90.8%], 86.5% (95% PPI = 81.8–90.8%) and 79.1% (95% PPI =73.4–84.5%), respectively. The Sp for anti-cat ELISA and HI test were close to the prior amounts with median values of 64.6% (95% PPI = 47.5–80.0%) and 54.1% (95% PPI = 30.0–76.2%), respectively. The Se values for anti-tiger ELISA were similar to the prior median value of 57.2% (95% PPI = 41.7–71.8%). Lastly, posterior estimates for the prevalence of tiger antibodies against FPV vaccine were higher than the prior estimate with a median value of 94.6% (95% PPI = 87.6–98.4%).

**Table 3:** Posterior estimates for sensitivity and specificity of indirect ELISA and HI tests, and the prevalence

Diagnostic tests	Parameters	Posterior estimates	
		Median (%)	95% PPI^a^
	Prevalence	94.6	87.6–98.4
ELISA (anti-cat)	Sensitivity	86.7	82.0–90.8
	Specificity	64.6	47.5–80.0
ELISA (anti-tiger)	Sensitivity	86.5	81.8–90.8
	Specificity	57.2	41.7–71.8
HI	Sensitivity	79.1	73.4–84.5
	Specificity	54.1	30.0–76.2

^a^*95% PPI* 95% posterior probability interval

The conditional dependence among two ELISA tests and the HI test was low among both seropositive and seronegative tigers, with probability intervals of the conditional covariance being 0. The conditional independent model, which did not include a covariance term among all three tests, had a higher deviance information criterion (DIC) value than that of the conditional dependent model (120 vs. 58, respectively). Therefore, the conditional dependent model was selected as the final model.

The model was converged properly and any autocorrelation was eliminated after omitting the first 10,000 iterations (Additional file 1: Figure S1). In the sensitivity analysis, no major changes (changes in median or 95% probability percentiles > 25%) were observed in the posterior sensitivity estimates for all three tests, while specificity estimations of both ELISA tests were used as the prior values for any parameter when non-informative distributions were applied. This result was interpreted as positive evidence of model robustness. In contrast, a change in the posterior estimates of specificity for the HI test was observed with a higher estimated posterior specificity (from 54.1 to 72.9%) when a non-informative prior value was used. Therefore, it is suggested that the prior values of this parameter had a stronger influence on the results of the model.

## Discussion

Tigers are the largest cat species and an important member of the ecosystem. Previous studies have identified the FPV infection in several populations of tigers [6, 7]. This evidence indicates that tigers appear to be susceptible to FPV infection. Vaccination against FPV among wild felid species has long been practiced in zoos worldwide, but few studies have assessed tiger immune response post-vaccination. Considering serum antibody titer, it has been shown to be useful for the determination of immune responses against viruses or vaccinations. Moreover, it is very important to predict the required frequency for the administration of the vaccine [13–15]. Therefore, an in-house indirect ELISA was developed in this study.

FPV is a non-enveloped single-strand deoxyribonucleic acid (ssDNA) virus that is classified in the family *Parvovirida*e, genus *Parvovirus*. The parvoviruses are comprised of small viral particles containing two major open reading frames (ORF), which encode non-structural (NS) proteins and capsid proteins including VP1 (10%) and VP2 (90%) [9]. VP2 protein is a major structural protein of the parvovirus. It has been highly conserved and is involved with receptor recognition and nuclear translocation [16]. Amino acid values at positions of 80, 564, and 568 are important for efficient viral replication in cats and are conserved among all canine parvovirus (CPV) and FPV viruses [17–19]. Previous studies have identified the capsid protein VP2 of FPV as a coating antigen for ELISA in the detection of antibodies against feline panleukopenia in cats [12]. In addition, the VP2 subunit protein exhibited good antigenicity in ELISA development for the detection of antibodies against parvovirus in minks causing Aleutian Mink disease with high sensitivity and specificity [20]. On that basis, the amino acids 545–585 of VP2, which covered the epitope region and were specific to the FPV, were selected and employed as an antigen in an indirect ELISA test in this study. The results showed that an in-house ELISA test using the VP2 peptide had better sensitivity and specificity when compared with the HI test. Therefore, it was found to be preferable over the HI test for the serological screening of tiger antibodies against FPV vaccine or infection.

ELISA and HI tests have been developed to assess antibody titers against feline parvovirus infections in cats [21, 22]. However, the specificity of the serum neutralizing (SN) test is the highest among viral serologic testing [23]. Since the SN test is considered time-consuming and costly, the HI test is being widely used to determine antibody titer against FPV [14, 23–25]. The ELISA test has been recognized as a sensitive and reliable method to determine humoral antibody response against parvovirus, possibly allowing for the calculation of the time that vaccinations can be performed [14, 26]. Bayesian latent class analysis is a statistical procedure for estimating the sensitivity and specificity of a diagnostic test in the absence of a gold standard assay. A Bayesian model has been used to estimate the sensitivity and specificity of various diagnostic techniques [27–31]. In this study, a Bayesian model was performed to estimate the sensitivity and specificity of an in-house indirect ELISA with two HRP-conjugated antibodies, and these values were compared to those of the HI test. The estimated sensitivity of anti-cat ELISA (86.7%) and anti-tiger ELISA (86.5%) tests were higher than that of the HI test (79.1%). In addition, the estimated specificity values of ELISA on anti-cat and anti-tiger antibodies (64.6 and 57.2%, respectively) were higher than that of the HI test (54.1%) as well. Therefore, the developed in-house ELISA appears to be more efficient for the screening and monitoring of antibody levels against FPV vaccination or infection in tigers.

The sensitivity and specificity of a test are usually determined through the detection of positive and negative serum samples by comparison with a gold standard test. In this study, there was no gold standard available to determine the status of the disease or antibody titer. Therefore, the accuracy of newly developed diagnostic tests should be estimated appropriately in the absence of a gold standard. Recently, the Bayesian approach has been successfully applied to estimate the degrees of sensitivity and specificity in the absence of a gold standard [27, 28, 30, 31]. The advantage of having access to Bayesian statistical inferences is the ability to simply model all of the uncertainties of the parameters with regard to probability [27]. Ultimately, it is not expected that the precise values would be known unless the quality of the prior information was extremely precise. Consequently, the term of uncertainty can be widely interpreted. An event can be uncertain by virtue of being intrinsically unpredictable due to random variations or imperfect knowledge. Therefore, scientific information about unknown parameters can be incorporated into the model through the specification of a prior joint probability distribution determination for all parameters of interest coupled with the truly known data. This is done in order to obtain an accurate understanding of posterior distribution. Nevertheless, prior scientific knowledge about unknown parameters is, in principle, subjective for analysis even with expert opinion [27]. Thus, the most important consideration in the use of prior information is to ensure that the prior distribution honestly reflects genuine information, not personal bias, prejudice and superstition. The prior information should be based on sound evidence and reasoned judgments. The posterior estimates for the prevalence of tiger antibodies against FPV were significantly higher than the prior estimates. This finding can be explained by the fact that most of the blood samples in this study were collected from vaccinated tigers. Thus, posterior estimates of the prevalence of tiger antibodies were higher than initially expected. For the sensitivity analysis, an important change was observed only in terms of the specificity of the HI test when non-informative prior distributions were applied in the model. The model was found to be sensitive to the prior selections for some parameters. There were no previous reports on the performance of the HI test for FPV detection in tigers. Consequently, prior values of the HI test were estimated based on expert opinions due to a lack of available information. This suggests that the HI test may be associated with higher specificity values than initially expected.

## Conclusions

An in-house indirect ELISA test for the monitoring of tiger antibody levels against the feline panleukopenia vaccine has been successfully developed in this study. Specifically, 1 ng per well of capsid protein VP2 synthetic peptide was used as a coating antigen. The optimal dilutions of the primary antibody (tiger serum) and secondary antibody (HRP-conjugated antibody) were 1:100, 1:2000 for the anti-tiger IgG antibody and 1:200, 1:1000 for the anti-cat IgG antibody. The calculated cut-off values of the indirect ELISA anti-tiger and anti-cat antibodies were 0.100 and 0.400, respectively. The Bayesian values of Se and Sp for the indirect ELISA anti-tiger antibodies were 86.2 and 57.5%, while they were 86.8 and 64.2% for the indirect ELISA anti-cat, respectively. Importantly, the Se and Sp values for the indirect ELISA were higher than those of the HI test (Se = 79.2%, Sp = 54.0%). These findings suggest that the in-house indirect ELISA test that was successfully developed in this study could be used as a potentially valuable serological tool for the effective detection of tiger antibodies.

## Methods

### Production of rabbit anti-tiger IgG polyclonal antibody

Purified Bengal tiger IgG was obtained from a previous study [32]. New Zealand white rabbits (Mlac:NZW, National Laboratory Animal Center, Mahidol University, Nakhon Pathom, Thailand) were weekly subcutaneously immunized with purified tiger IgG (1 mg/dose.1 ml) formulated with Montanide™ ISA 206 VG (Seppic, Paris, France; 1:1 v/v, 100 μg/ml) for 4 weeks. The rising titer of rabbit anti-tiger IgG was measured using indirect ELISA [32]. Due to the blood collection of rabbits, the generalized anesthesia was done by intravenous injection of pentobarbitone sodium (Nembutal, 20 mg/kg). The blood collection was done by using a 1-in. long, 18-gauge needle for penetrating to jugular vein. Blood was taken until the volume reached 100 ml, then the rabbits were euthanized by intravenous injection of overdosage pentobarbitone sodium (Nembutal, 90 mg/kg). Rabbit antisera were then purified using a Melon™ Gel IgG Purification Kit following the manufacturer’s instructions. Concentrations of purified rabbit anti-tiger IgG polyclonal antibody were measured using an bicinchoninic acid (BCA) protein assay (BCA protein assay kit; Pierce, Rockford, IL, USA) according to the manufacturer’s instructions. The purified concentrations were then stored at − 20 °C for further analysis.

### Preparation of HRP-conjugated rabbit anti-tiger IgG antibody

The two-step glutaraldehyde method was used to couple HRP with rabbit anti-tiger IgG as has been previously described by Chansiw et al. (2008) [33]. Briefly, 2 mg of HRP (Sigma-Aldrich, St. Louis, Missouri, USA) were dissolved in 200 μl of 0.1 M potassium phosphate buffer (pH 6.8) in 1.25% glutaraldehyde 200 μl and incubated at 4 °C for 18 h using an end-over-end rotator. Excess free glutaraldehyde was removed by 10 K Vivaspin® 500 (Sartorius Stedim Biotech Gmbh, Goettingen, Germany) and the specimens were centrifuged at 10,000 rpm for 30 min. They were then resuspended with 200 μl of 0.1 M sodium carbonate-bicarbonate buffer (pH 9.5) with 30% sucrose and collected in a microcentrifuge tube. Micro Bio-Spin™ Chromatography Columns (Bio-Rad Laboratories, Hercules, CA, USA) were used for the process of buffer exchange to achieve the appropriate buffer for the antibody according to the manufacturer’s instructions. Subsequently, 500 μl of 0.15 M NaCl were added to the column, and the column was then centrifuged at 1000×g for 1 min and washed three times. Rabbit anti-tiger IgG 1 mg/100 μl was carefully added directly to the center of the column, and the column was then centrifuged at 1000×g for 4 min. Rabbit anti-tiger IgG (1 mg/100 μl) was mixed with HRP (2 mg/200 μl) and incubated overnight at 4 °C using an end-over-end rotator. Additionally, 15 μl of 2 M glycine were added and the specimens were centrifuged by 50 K Amicon® Ultra-15 (Merck Millipore Ltd., Cork, Ireland) at 3400 rpm for 30 min to eliminate the excess HRP and other buffers. Subsequently, 100 μl of the phosphate-buffered saline (PBS, pH 7.2) was resuspended. Next, 500 μl of PBS buffer (pH 7.2) were added to the Micro Bio-spin® Chromatography Column for buffer exchange. The column was then centrifuged at 1000×g for 1 min and washed three times. HRP-IgG solution was then carefully added to the column and it was centrifuged at 1000×g for 4 min in order to allow the solution to collect in a microcentrifuge tube. HRP-conjugated rabbit anti-tiger IgG was obtained and then stored at − 20 °C for further analysis.

### Determination of avidity and specificity of tiger IgG and rabbit anti-tiger IgG polyclonal antibodies by western blot analysis

Immunochemical analysis of purified protein was done by western blot technique according to the method of Towbin et al. (1979) [34] with some modifications. Briefly, the purified tiger IgG was separated by sodium dodecyl sulfate polyacrylamide gel electrophoresis (SDS-PAGE) according to the method described by Laemmli (1970) [35]. Samples were prepared in sample buffer and boiled at 95 °C for 5 min and then analyzed on 10% SDS-PAGE gel slabs in a mini-slab apparatus (Bio-Rad Laboratories, Hercules, CA, USA). The protein on SDS-PAGE gel was electrically transferred onto a nitrocellulose membrane (Merck Millipore™, Merck KGaA©, Darmstadt, DEU). The blotting time was set at 60 min at a constant voltage of 10 V. The membrane was incubated with blocking buffer (5% skim milk in PBS) for 1 h at room temperature with gentle shaking. After being washed three times with washing buffer (PBS containing 0.05% Tween® 20; PBST), the membrane was probed with HRP-conjugated rabbit anti-tiger IgG (1:500 dilution) obtained from the previous step and HRP-conjugated goat anti-cat IgG (1:2000 dilution; KPL, Gaithersburg, MD, USA) antibodies, separately. The membrane was then incubated with gentle shaking at room temperature for 1 h and washed three times. Finally, the reactions were visualized using a solution containing 3,3′-diaminobenzidine (DAB; Invitrogen, Carlsbad, CA, USA) with hydrogen peroxide (H_2_O_2_; Merck, Germany).

### Animals

All of the tigers included in this study belonged to the Tiger Kingdom, Mae Rim, Chiang Mai, Thailand. During the course of this study, the tigers were fed, housed and managed according to the official Tiger Care Manual [11]. All sampling procedures were monitored and controlled under the supervision of a veterinarian. After the study, all of the tigers, which were deemed to be a protected species of wildlife in Thailand, were kept and looked after at the Tiger Kingdom, Mae Rim and at the Tiger Kingdom Learning Centre, Mae Tang, Chiang Mai.

### Vaccination and blood sampling

Forty-three tigers were included in this study. All tigers were vaccinated with an inactivated vaccine (Fel-O-Vax® 4 Vaccine; Boehringer Ingelheim Vetmedica, Inc. St. Joseph, MO, USA) according to the vaccination protocol described in the Tiger Care Manual [11]. Blood samples were collected from the saphenous vein after administering anesthesia. A blood sample was taken from each tiger prior to the vaccinations and every 3 months for 1 year. Sera were prepared by centrifugation and stored at − 20 °C for further analysis.

### Hemagglutination inhibition (HI) test

Tiger sera were tested using the HI test following the method that had been previously described [36]. An HI titer value that was higher than 1:20 was considered seropositive.

### Capsid protein or viral protein 2 (VP2) subunit antigen prediction and synthesis

The VP2 subunit antigen was predicted based on the amino acid sequence accession number ABN70938.1 using the linear and conformational epitope prediction software as has been described in the previous report [37]. The selected amino acid sequence (WNPIQQMSINVDNQFNYVPNNIGAMKIVYEKSQLAPRKLY) was then further synthesized (Genscript, Piscataway, New Jersey, USA).

### In-house indirect ELISA

The reaction was performed in 96-well plates (Nunc-Immuno™ MaxiSorp™; Sigma-Aldrich) in duplicate. Each well was coated with an optimal concentration of synthetic VP2 capsid protein with coating buffer (0.05 M carbonate bicarbonate buffer, pH 9.6). Each plate was incubated overnight at 4 °C and then washed three times with 200 μl of washing buffer (PBST, pH 7.2). Non-specific binding sites were blocked with 100 μl per well of blocking buffer (1% skim milk in PBS) for 1 h at 25 °C. After being washed three times with PBST, 100 μl of tiger serum that was diluted in blocking buffer was added to each well and the plate was incubated for 1 h at 25 °C. After being washed three times with PBST, 100 μl of either HRP-conjugated goat anti-cat IgG (KPL) or HRP-conjugated rabbit anti-tiger antibody diluted in blocking buffer were added to each well. The reaction was performed for 1 h at 25 °C. After completion, any excess antibody was washed out with PBST over the course of three washings. One hundred μl of tetramethylbenzidine (TMB; KPL) was added to each well as a substrate. The reaction was performed in the dark for 15–30 min at room temperature before being stopped by adding 50 μl of 2 N H_2_SO_4_. The absorbance was measured at a wavelength of 450 nm using an automatic ELISA plate reader (AccuReader; Metertech, Taipei, Taiwan). The results were expressed as an OD value. The assay was carried out in two separate trials in order to obtain reproducible data.

### Optimization of an in-house indirect ELISA

Checkerboard titration was performed according to Crowther (2009) [38] to determine the optimal dilutions of the sera and conjugated antibody. Pooled vaccinated sera and pooled non-vaccinated sera were analyzed according to the ELISA method as has been previously described and ranged from 1:100 to 1:500 dilutions. The HRP-conjugated antibody varied from 1:1000 to 1:4000 dilutions. The optimal dilutions of the serum and HRP-conjugated antibody were defined as those for which the ratio was the greatest between the vaccinated and non-vaccinated sample OD values.

### Calculation of cut-off value

The cut-off value was obtained by employing an OD value at a wavelength of 450 nm and was calculated from the mean plus 3 value of the standard deviations (SD) of the non-vaccinated group [38]. For further interpretation, any tiger sera with an OD value of more than or equal to the cut-off value were classified as seropositive. Tiger sera with an OD value that was less than the cut-off value were classified as seronegative.

### Determination of sensitivity and specificity

A latent class analysis was performed using a Bayesian model to determine the sensitivity and specificity of two indirect ELISA tests and the HI test. Since all three tests are based on the detection of an antibody response, the results were considered conditionally dependent upon each other [39]. Therefore, a Bayesian model for three diagnostic tests was implemented in one population in order to determine the sensitivity and specificity of each test.

Prior information on test performance and the prevalence of antibodies was introduced into the analysis using probability distributions (prior to distribution). As a result of a lack of prior information on three tests, the sensitivity and specificity of the prior values for indirect ELISA and HI tests were modeled as beta distributions based on three expert opinions. Prior values for the prevalence of antibodies against FPV vaccine in tigers in Chiang Mai were also selected based on opinions from three zoo and wildlife experts due to the absence of published information on this point. Prior values used for analysis (sensitivity, specificity, and prevalence) are listed in Table 4. All analyses were implemented in JAGS 3.4.0 [40] via the rjags and R2jags packages [41, 42] obtained from R 3.2.2 software [43]. Posterior distributions were computed after 100,000 iterations of models with the first 10,000 being discarded as the burn-in phase.

**Table 4:** Prior values used for analysis in this study

Diagnostic tests	Parameters	Prior values	
		Mode (%)	95% CI^a^
	Prevalence	50	< 80
ELISA (anti-cat)	Sensitivity	75	> 63
	Specificity	66	> 50
ELISA (anti-tiger)	Sensitivity	68	> 53
	Specificity	57	> 43
HI	Sensitivity	56	> 40
	Specificity	50	> 30

^a^*95% CI* 95% confidence interval

The model convergence was assessed by visual inspection of the Gelman-Rubin diagnostic plots [44, 45] using three sample chains with different initial values. The goodness of fit of the models was determined using DIC [46], and the number of effectively estimated parameters (pD) [47] served as the calibrating parameters. The model sensitivity analysis was performed to assess the influence of prior information and the assumption of conditional dependence between the results of two ELISA tests and the HI test on the posterior estimates [27, 48]. These analyses were performed by replacing each prior value with a non-informative uniform 0–1 distribution and by comparing the DIC values between models with and without the covariance term [27].

## Supplementary information

**Additional file 1 : Figure S1.** Western blotting analysis of the avidity and specificity of rabbit anti-tiger IgG polyclonal antibody against tiger IgG. Lane 1–4 were loaded with the new stock of purified tiger IgG. Lane 5–7 were loaded with the previous stock of purified tiger IgG. Lane 1 was probed with rabbit HRP-anti-cat IgG. Lane 2–7 were probed with rabbit HRP-anti-tiger IgG. Note that the lane 1 and lane 4 were selected to construct the new figure for manuscript.

## Data Availability

All relevant data in this study are available from the corresponding author upon reasonable request.

## Abbreviations

BCA: Bicinchoninic acid
CPV: Canine parvovirus
DIC: Deviance information criterion
DAB: 3,3′-diaminobenzidine
ELISA: Enzyme-linked immunosorbent assay
FPV: Feline panleukopenia virus
HI: Hemagglutination-inhibition
HRP: Horseradish peroxidase
H_2_O_2_: Hydrogen peroxide
IUCN: International Union for Conservation of Nature
IgG: Immunoglobulin G
MLV: Modified live vaccines
NS: Non-structural
pD: Number of effectively estimated parameters
ORF: Open reading frames
OD: Optical density
PBS: Phosphate-buffered saline
PBST: Phosphate-buffered saline containing 0.05% Tween® 20
PPI: Posterior probability interval
SN: Serum neutralizing
S/N: Signal to noise
ssDNA: single-strand deoxyribonucleic acid
SDS-PAGE: Sodium dodecyl sulfate polyacrylamide gel electrophoresis
SD: Standard deviations
H_2_SO_4_: Sulfuric acid
TMB: Tetramethylbenzidine
VP2: Viral protein 2

## References

[1] Barker IK, Povey RC, Voigt DR (1983). Response of mink, skunk, red fox and raccoon to inoculation with mink virus enteritis, feline panleukopenia and canine parvovirus and prevalence of antibody to parvovirus in wild carnivores in Ontario. Can J Com Med.

[2] Kruse BD, Unterer S, Horlacher K, Sauter-Louis C, Hartmann K (2010). Prognostic factors in cats with feline panleukopenia. J Vet Int Med.

[3] Scott FW, Csiza CK, Gillespie JH (1970). Maternally derived immunity to feline panleukopenia. J Am Vet Med Assoc.

[4] Steinel A, Parrish CR, Bloom ME, Truyen U (2001). Parvovirus infections in wild carnivores. J Wildl Dis.

[5] Stuetzer B, Hartmann K (2014). Feline parvovirus infection and associated diseases. Vet J.

[6] Duarte MD, Barros SC, Henriques M, Fernandes TL, Bernardino R, Monteiro M (2009). Fatal infection with feline panleukopenia virus in two captive wild carnivores (*Panthera tigris* and *Panthera leo*). J Zoo Wildl Med.

[7] Dissanayake DRA, Silva ID, Gamage S, Sonnadara D, Bandara MRBN, Alokabandara SS (2017). Feline panleukopenia virus infection in a captive-bred Bengal tiger (*Panther tigris tigris*) and a leopard (*Panthera pradus*). S L Vet J.

[8] Goodrich J, Lynam A, Miquelle D, Wibisono H, Kawanishi K, Pattanavibool A, et al. *Panthera tigris*. The IUCN Red List of Threatened Species 2015:e.T15955A50659951. 2015. 10.2305/IUCN.UK.2015-2.RLTS.T15955A50659951.en. Accessed 26 De 2019.

[9] Truyen U, Parrish CR (2013). Feline panleukopenia virus: its interesting evolution and current problems in immunoprophylaxis against a serious pathogen. Vet Microbiol.

[10] Lamberski N, Miller RE, Fowler ME (2015). Felidae. Fowler’s zoo and wild animal medicine, Volume 8.

[11] AZA Tiger Species Survival Plan®. Tiger Care Manual. Association of Zoos and Aquariums. 2016. https://assets.speakcdn.com/assets/2332/tiger_care_manual_2016.pdf. Accessed 28 Dec 2019.

[12] Yang S, Xia X, Qiao J, Liu Q, Chang S, Xie Z (2008). Complete protection of cats against feline panleukopenia virus challenge by a recombinant canine adenovirus type 2 expressing VP2 from FPV. Vaccine..

[13] DiGangi BA, Gray LK, Levy JK, Dubovi EJ, Tucker SJ (2011). Detection of protective antibody titers against feline panleukopenia virus, feline herpesvirus-1, and feline calicivirus in shelter cats using a point-of-care ELISA. J Feline Med Surg.

[14] Lappin MR, Andrews J, Simpson D, Jensen WA (2002). Use of serologic tests to predict resistance to feline herpesvirus 1, feline calicivirus, and feline parvovirus infection in cats. J Am Vet Med Assoc.

[15] Risi E, Agoulon A, Allaire F, Le Dréan-Quénec’hdu S, Martin V, Mahl P (2012). Antibody response to vaccines for rhinotracheitis, caliciviral disease, panleukopenia, feline leukemia, and rabies in tigers (*Panthera tigris*) and lions (*Panthera leo*). J Zoo Wildl Med..

[16] Tu M, Liu F, Chen S, Wang M, Cheng A (2015). Role of capsid proteins in parvoviruses infection. Virol J.

[17] Truyen U, Agbandje M, Parrish CR (1994). Characterization of the feline host range and a specific epitope of feline panleukopenia virus. Virology..

[18] Truyen U, Evermann JF, Vieler E, Parrish CR (1996). Evolution of canine parvovirus involved loss and gain of feline host range. Virology..

[19] Steinel A, Munson L, Van Vuuren M, Truyen U (2000). Genetic characterization of feline parvovirus sequences from various carnivores. J Gen Virol.

[20] Ma F, Zhang L, Wang Y, Lu R, Hu B, Lv S (2016). Development of a peptide ELISA for the diagnosis of Aleutian mink disease. PLoS One.

[21] Fiscus SA, Mildbrand MM, Gordon JC, Teramoto YA, Winston S (1985). Rapid enzyme-linked immunosorbent assay for detecting antibodies to canine parvovirus. Am J Vet Res.

[22] Hofmann-Lehmann R, Fehr D, Grob M, Elgizoli M, Packer C, Martenson JS (1996). Prevalence of antibodies to feline parvovirus, calicivirus, herpesvirus, coronavirus, and immunodeficiency virus and of feline leukemia virus antigen and the interrelationship of these viral infections in free-ranging lions in East Africa. Clin Diagn Lab Immunol.

[23] Soma T, Ohta K, Yamashita R, Sasai K (2019). Anti-feline panleukopenia virus serum neutralizing antibody titer in domestic cats with the negative or low hemagglutination inhibition antibody titer. J Vet Med Sci.

[24] Jakel V, Cussler K, Hanschmann KM, Truyen U, König M, Kamphuis E (2012). Vaccination against feline panleukopenia: implications from a field study in kittens. BMC Vet Res.

[25] Thompson H, Macartney L, McCandlish IA, Cornwell HJ (1985). Measurement of antibodies after parvovirus vaccination. Vet Rec.

[26] Mylonakis M, Kalli I, Rallis T (2016). Canine parvoviral enteritis: an update on the clinical diagnosis, treatment, and prevention. Vet Med.

[27] Branscum AJ, Gardner IA, Johnson WO (2005). Estimation of diagnostic-test sensitivity and specificity through Bayesian modeling. Prev Vet Med.

[28] Poolperm P, Varinrak T, Kataoka Y, Tragoolpua K, Sawada T, Sthitmatee N (2017). Development and standardization of an in-house indirect ELISA for detection of duck antibody to fowl cholera. J Microbiol Methods.

[29] Schlichting D, Nöckler K, Bahn P, Luge E, Greiner M, Müller-Graf C (2015). Estimation of the sensitivity and specificity of a *Leptospira spp.* in-house ELISA through Bayesian modelling. Int J Med Microbiol.

[30] Singhla T, Boonyayatra S, Chulakasian S, Lukkana M, Alvarez J, Sreevatsan S (2019). Determination of the sensitivity and specificity of bovine tuberculosis screening tests in dairy herds in Thailand using a Bayesian approach. BMC Vet Res.

[31] Tankaew P, Singh-La T, Titaram C, Punyapornwittaya V, Vongchan P, Sawada T (2017). Evaluation of an in-house indirect ELISA for detection of antibody against haemorrhagic septicemia in Asian elephants. J Microbiol Methods.

[32] Areewong C, Sangchantip R, Rungphattanachaikul S, Rittipornlertrak A, Fhaikruae I, Wongkalasin W (2019). Production and characterization of polyclonal antibody against Bengal tiger (*Panthera tigris tigris*) immunoglobulin G. J Appl Anim Res.

[33] Chansiw N, Pornsilapathip J, Vongchan P (2008). Production of horseradish peroxidase conjugated monoclonal anti-heparan sulfate proteoglycans antibody for immunologic assay. Bull Chiang Mai Assoc Med Sci.

[34] Towbin H, Staehelin T, Gordon J (1979). Electrophoretic transfer of proteins from polyacrylamide gels to nitrocellulose sheets: procedure and some applications. Proc Natl Acad Sci.

[35] Laemmli UK (1970). Cleavage of structural proteins during the assembly of the head of bacteriophage T4. Nature..

[36] Nakamura K, Ikeda Y, Miyazawa T, Tohya Y, Takahashi E, Mochizuki M (2001). Characterization of cross-reactivity of virus neutralizing antibodies induced by feline panleukopenia virus and canine parvoviruses. Res Vet Sci.

[37] Rittipornlertrak A, Nambooppha B, Simking P, Punyapornwithaya V, Tiwananthagorn S, Jittapalapong S (2017). Low levels of genetic diversity associated with evidence of negative selection on the *Babesia bovis* apical membrane antigen 1 from parasite populations in Thailand. Infect Genet Evol.

[38] Crowther JR (2009). The ELISA guidebook.

[39] Gardner IA, Stryhn H, Lind P, Collins MT (2000). Conditional dependence between tests affects the diagnosis and surveillance of animal diseases. Prev Vet Med..

[40] Plummer M. JAGS: A program for analysis of Bayesian models using Gibbs sampling. Proceedings of the 3rd International Workshop on Distributed Statistical Computing; Vienna, Austria. 2003. http://www.ci.tuwien.ac.at/Conferences/DSC-2003/. Accessed 20 Dec 2019.

[41] Su U, Yajima M (2015). R2jags: Using R to run “JAGS” (R package version 0.5–7).

[42] Plummer M, Stukalov A, Denwood M (2016). Bayesian graphical models using MCMC: package ‘rjags’.

[43] R Core Team (2015). R: A language and environment for statistical computing.

[44] Gelman A, Rubin DB (1992). Inference from iterative simulation using multiple sequences. Stat Sci.

[45] Brooks SP, Gelman A (1998). General methods for monitoring convergence of iterative simulations. J Comput Graph Stat.

[46] Spiegelhalter DJ, Best NG, Carlin BP, Van Der Linde A (2002). Bayesian measures of model complexity and fit. J Royal Stat Soci Ser B.

[47] Berkvens D, Speybroeck N, Praet N, Adel A, Lesaffre E (2006). Estimating disease prevalence in a Bayesian framework using probabilistic constraints. Epidemiology..

[48] Rahman AKMA, Saegerman C, Berkvens D, Fretin D, Gani MO, Ershaduzzaman M (2013). Bayesian estimation of true prevalence, sensitivity and specificity of indirect ELISA, rose Bengal test and slow agglutination test for the diagnosis of brucellosis in sheep and goats in Bangladesh. Prev Vet Med..

