# Supplementary figures and images for: Evaluation of an in-house indirect enzyme-linked immunosorbent assay of feline panleukopenia VP2 subunit antigen in comparison to hemagglutination inhibition assay to monitor tiger antibody levels by Bayesian approach
Source: BMC Vet Res. 2020 Aug 6;16:275. doi: 10.1186/s12917-020-02496-z (PMC7409676; doi:10.1186/s12917-020-02496-z)

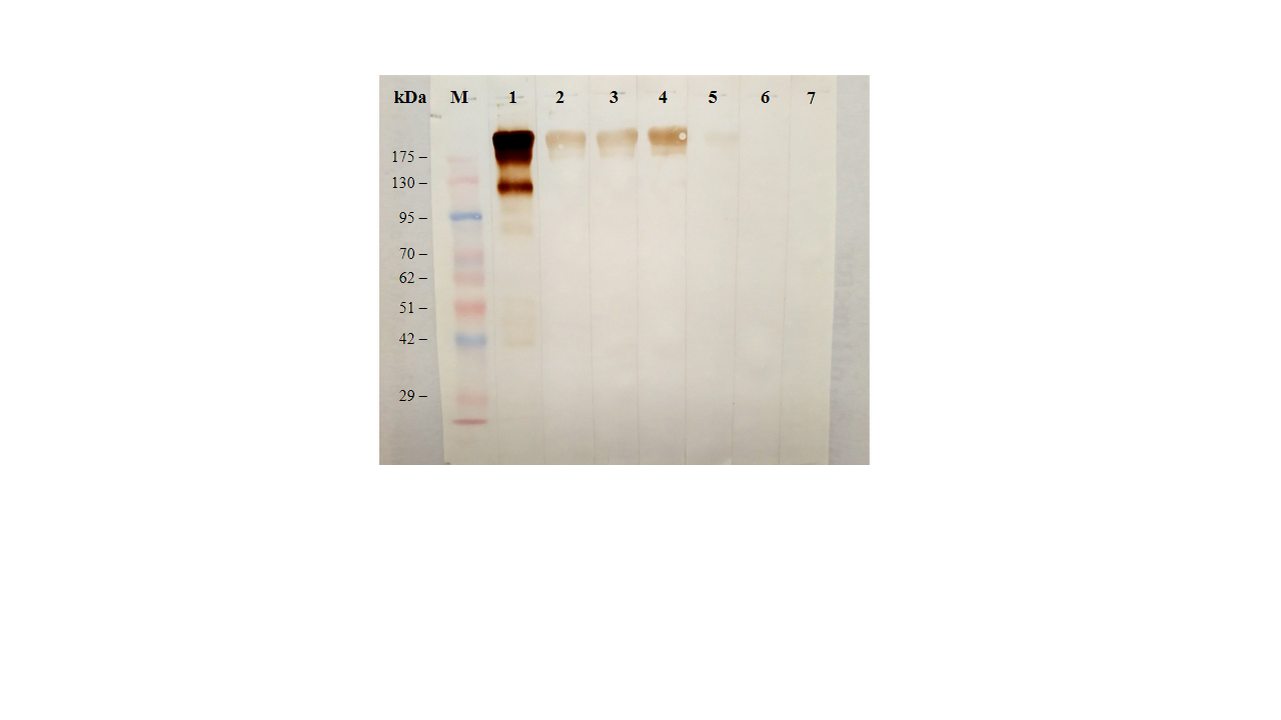

Supplement: Supplementary file 1 — Additional file 1 : Figure S1. Western blotting analysis of the avidity and specificity of rabbit anti-tiger IgG polyclonal antibody against tiger IgG. Lane 1–4 were loaded with the new stock of purified tiger IgG. Lane 5–7 were loaded with the previous stock of purified tiger IgG. Lane 1 was probed with rabbit HRP-anti-cat IgG. Lane 2–7 were probed with rabbit HRP-anti-tiger IgG. Note that the lane 1 and lane 4 were selected to construct the new figure for manuscript. [file 12917_2020_2496_MOESM1_ESM.tif]
